# Effects of Long-Term Administration of Ginsenosides on the Structure of Intestinal Microflora and the Absorption and Utilization of Saponins in Rats

**DOI:** 10.3390/metabo16060352

**Published:** 2026-05-25

**Authors:** Guoliang Dou, Xinyue Bi, Nuoyan Wang, Shuhang Li, Yu Huang, Shirui Lu, Zixin Wang, Wenxiu Ji, Ye Hong, Weiwei Dong

**Affiliations:** College of Agricultural, Yanbian University, Park Road 977, Yanji 133002, China

**Keywords:** ginsenosides, gut microbiota, pharmacokinetics

## Abstract

**Highlights:**

**What are the main findings?**

- The *Lactobacillus* proportion in the PGE group increased by 17.78% ± 4.37% compared to CK (*p* < 0.05).
- The PGE group showed significantly higher AUC and C_max_ of ginsenoside compound K than the CK group.

**What are the implications of the main findings?**

- Long-term oral administration of ginsenosides can increase the levels of beneficial probiotic bacteria in the intestines.
- Long-term oral administration of ginsenosides (30 days) can enhance their absorption in the body.

**Abstract:**

**Background:** Ginsenosides are active natural compounds with diverse effects, and their interaction with the gut microbiota can influence microbial composition and abundance, though the long-term effects remain unclear. **Methods:** This study examines the impact of long-term oral ginsenoside administration on gut microbiota composition and structure in rats, as well as its pharmacokinetics. Twenty healthy male Wistar rats were divided into a control group (CK, receiving distilled water) and a ginsenoside treatment group (PGE, 100 mg/kg) for 30 days. Fecal samples were analyzed using 16S rRNA high-throughput sequencing on the Illumina HiSeq platform to assess microbial diversity. Concurrently, liquid chromatography–tandem mass spectrometry (LC-MS/MS) was utilized to determine the concentrations of ginsenosides in the serum and to investigate their pharmacokinetic properties (*p* < 0.05). **Results:** The results indicated that the α-diversity indices of the gut microbiota in the PGE group were significantly higher than in the CK group, suggesting that ginsenosides enhance microbial richness and diversity (*p* < 0.05). At the phylum level, the relative abundance of *Firmicutes* in the PGE group increased by 10.6% ± 2.72%, while that of *Bacteroidetes* decreased by 11.5% ± 3.18%; at the genus level, the proportion of *Lactobacillus* genus rose by 17.78% ± 4.37% (*p* < 0.05). Pharmacokinetic analysis revealed that the area under the concentration–time curve (AUC) and maximum concentration (C_max_) of ginsenosides were significantly higher in the PGE group than in the CK group. **Conclusions:** Chronic oral administration of ginsenosides improves their absorption and utilization through gut microbiota modulation, offering experimental evidence for deeper insight into ginsenoside–microbe interactions.

## 1. Introduction

Often termed the “second genome” of humans, the gut microbiota is essential for regulating various vital physiological functions and overall health. It is involved in key processes such as metabolism, neurotransmission, and immune modulation [1,2,3]. In normal physiological circumstances, a dynamic ecological equilibrium exists among the gut microbiota, the host organism, and the surrounding environment. If this homeostasis is disrupted, it can lead to symptoms such as bloating, diarrhea, and constipation in the short-term; long-term imbalance may result in various chronic metabolic diseases, including obesity, diabetes, and cardiovascular diseases [4,5]. Interactions between the host and the gut microbiota result in the production of a variety of metabolites. These include those generated from undigested dietary components, such as short-chain fatty acids, as well as metabolites originating from microorganisms and endogenous substances [6,7]. Research indicates that the gut microbiota regulates the host immune system directly or indirectly by producing active molecules such as short-chain fatty acids (SCFAs), tryptophan metabolites, and polysaccharide derivatives, influencing inflammatory responses and the tumor microenvironment, thereby providing new targets for the treatment of various diseases [8,9,10].

Traditional Chinese medicine and its active components, due to their multi-component and multi-target characteristics, not only provide metabolic substrates for the gut microbiota but also reshape the gut microecological balance through the interactions between microorganisms and the host, thereby influencing various physiological functions of the body [11,12]. Ginseng is one of the most widely used medicinal plants in the world and possesses various pharmacological activities. The active components of ginseng (such as saponins, polysaccharides, and dietary fibers) can act as prebiotics, helping to reduce inflammation in the gut, enhance intestinal barrier function, and promote the production of metabolites like short-chain fatty acids, selectively stimulating the proliferation of beneficial bacteria (such as *Lactobacillus*) and inhibiting harmful bacteria, thus improving the structure of the microbiota [13,14]. Ginsenosides are regarded as the primary active components that exert effects in ginseng, exhibiting antioxidant, anti-inflammatory, immunomodulatory, and anti-tumor properties [15]. Based on variations in the structure of aglycones, ginsenosides can be categorized into three main types: oleanolic acid type; protopanaxadiol type (PPD type), which includes compounds like Rb1, Rb2, and Rc; and protopanaxatriol type (PPT type), represented by Rg1, Re, and Rf. The structural diversity among these categories contributes to notable differences in their biological activities [16]. Natural ginsenosides are complex macromolecular compounds that are typically metabolized by the gut microbiota in the body to produce secondary metabolites that exert pharmacological activity. For example, after hydrolysis by gut bacteria (such as *Bacteroides*), Rb1 is deglycosylated to form the highly active and easily absorbable rare ginsenoside Compound K, thereby exerting anti-inflammatory and anti-tumor effects [17]. After hydrolysis by gut bacteria, Rf is deglycosylated to form the more pharmacologically active Rh1 [18]. Ginsenosides have the ability to enhance the growth of beneficial bacterial populations while simultaneously suppressing the proliferation of harmful bacteria, which contributes to the optimization of the gut microbiota composition. Ginsenoside Compound K has been found to significantly promote the growth of *Lactobacillus*, improving inflammatory bowel disease (IBD) [19].

In summary, a bidirectional regulatory relationship exists between ginsenosides and the gut microbiota: the gut microbiota transform ginsenosides into bioavailable active metabolites, which in turn modulate the composition of the gut microbiota, collectively contributing to health enhancement. This study selected 20 healthy male Wistar rats, which were randomly divided into a control group (CK, gavaged with distilled water) and a ginsenoside treatment group (PGE, gavaged with 100 mg/kg) for a 30-day intervention [20,21]. The environmental room temperature was maintained at 24 ± 1 °C, relative humidity at 50 ± 10%, and a standard lighting cycle of 12 h light and 12 h dark was followed. This study adopted 16S rRNA high-throughput sequencing technology to analyze the diversity of the gut microbiota and determine the pharmacokinetic characteristics of ginsenosides in rat blood, aiming to clarify the in vivo absorption and utilization extent of these compounds, and provide a theoretical basis for exploring their long-term effects on the composition of the gut microbiota as well as their roles in human absorption and utilization.

## 2. Materials and Methods

### 2.1. Materials

This study utilized a total of 20 healthy male Wistar rats aged 6 weeks, with body weights ranging from 180 to 200 g. All rats were SPF grade and purchased from Changchun Yisi Experimental Animal Co., Ltd., Changchun, China. The ginseng, aged four years, was sourced from Jilin Jian. Ginsenoside standards, including Re, Rg1, Rf, Rb1, Rc, Rb2, Rd, Rg2, F1, F2, and Compound K, were acquired from the Beijing National Standard Material Network, Beijing, China. Analytical grade phenol, penicillin, agarose, CTAB, Tris, SDS, and TEMED were purchased from Yanji Sanfu Chemical Co., Ltd., Yanji, China. Chromatographic-grade methanol, acetonitrile, water, and formic acid were acquired from Thermo Fisher Scientific in the United States. All other reagents used in the study were of analytical grade quality.

### 2.2. Extraction of Ginsenosides

The ginseng, aged four years, was sourced from Jilin Jian. The experiment used ginseng (*Panax ginseng*) as the raw material, grinding the ginseng roots and passing them through a 60-mesh sieve, then drying them in a 60 °C air-drying oven until constant weight was achieved. Precisely weigh 100 g of ginseng root powder and add it to 1000 mL of 70% ethanol solution at a liquid-to-material ratio of 1:10, and perform reflux extraction at 80 °C for two cycles, each lasting 3 h, combining the two extraction liquids for later use [22,23,24]. The extract was concentrated by rotary evaporation at 40 °C and then freeze-dried to obtain a crude extract of total ginsenosides, which was stored at −20 °C for future use. The extraction rate of ginsenosides is 5.8%.

### 2.3. Animal Experimental Design

All experimental procedures were conducted in accordance with the “Guidelines for the Care and Use of Laboratory Animals” by the National Institutes of Health (NIH) and were approved by the Animal Ethics Committee of the Animal Center of Yanbian University (Approval No: YD20250226030). After numbering the experimental animals, 20 healthy male Wistar rats were randomly divided into two groups of 10 each using a random number table: the control group (CK group) received distilled water orally every day, while the ginsenoside treatment group (PGE group) received ginsenoside administration daily for 30 consecutive days. These rats were adaptively fed for one week prior to the experiment. All rats were housed under identical conditions, with a controlled temperature of 22–24 °C, relative humidity of 50–70%, and a standard 12 h light/dark cycle. Food and water were provided ad libitum. The PGE group of rats was gavaged daily with 100 mg/kg of ginsenosides (containing 100 mg of ginsenosides per 10 mL of distilled water, with a gavage volume of 1 mL per 100 g of body weight), while the control group received an equal volume of distilled water daily. During the experiment, all rats were provided with the same standard diet and had ad libitum access to food and water. Uniform rearing and dietary conditions were strictly maintained across groups to exclude the confounding effects of dietary variation on gut microbiota structure and ginsenoside metabolism. Fecal samples from each group (5 samples per group) were collected on day 0 and day 30 of the experiment and stored at −80 °C for microbial analysis. On day 30, both the PGE and CK groups were administered 100 mg/kg of ginsenoside, and blood samples were collected from the tail vein at 0, 0.5, 1, 2, 4, 8, 12, and 24 h, followed by centrifugation at 5000 rpm to prepare plasma, which was stored at −20 °C for subsequent saponin content determination.

All rat gavage procedures were performed uniformly by experienced personnel to ensure consistency in operation, using a soft-tip gavage needle designed for rats to minimize injury. Daily administration was conducted between 9:00 and 10:00 a.m. on an empty stomach to exclude food interference with ginsenoside absorption. During the gavage process, each rat was kept in an upright position, and the operator slowly inserted the gavage needle along the esophagus, confirming no resistance before gradually injecting the solution.

Before blood collection from the tail vein, the tail was immersed in 40 °C warm water for 30 s to dilate the blood vessels. Blood was then collected using a 26G needle (Shanghai Hanming Medical Equipment Co., Ltd., Shanghai, China), with a single collection volume not exceeding 0.3 mL. The animals were housed in standard IVC cages, with two animals per cage, and provided with corncob bedding, wooden chew sticks, and paper nesting material for environmental enrichment. At the end of the experiment, the animals were euthanized by cervical dislocation after deep anesthesia with isoflurane.

### 2.4. LC-MS/MS Analysis of Metabolites

An Agilent 1260 series liquid chromatography system was utilized, equipped with an Eclipse Plus C18 column (4.6 mm × 250 mm, 5 μm; Agilent Technologies, Santa Clara, CA, USA). The mobile phase comprised water (designated as A) and acetonitrile (designated as B), employing a gradient elution protocol as follows: from 0 to 15 min, 23% to 30% B; from 15 to 34 min, 30% to 44% B; from 34 to 46 min, 44% to 68% B; from 46 to 61 min, 68% to 85% B; from 61 to 66 min, 85% to 80% B; and finally, from 66 to 73 min, 80% to 23% B. The flow rate was maintained at 0.5 mL/min, the column temperature was set at 25 °C, and the injection volume was 2 μL. Mass spectrometry detection was performed in positive ion electrospray ionization mode, with a capillary voltage of 4000 V, a gas flow rate of 13 L/min, an ion source temperature of 300 °C, and a full ion scan range of 100–1500. Qualitative and quantitative analysis of ginsenosides and their metabolites was conducted by detecting [M + Na]^+^ or [M − 2H_2_O + H]^+^ ions.

### 2.5. Calibration Curve for Ginsenosides in Rat Plasma

The quantitative assessment of ginsenoside concentrations was conducted utilizing the external standard approach. To prepare the calibration standards, working solutions were introduced into blank plasma obtained from rats. The resulting calibration standards encompassed ginsenoside concentrations of 50, 100, 200, 500, 1000, 2000, 5000, and 10,000 ng/mL. A calibration curve was constructed by graphing the ratio of the analyte peak areas to those of the internal standard against the concentrations of the spiked standards in plasma. This was accomplished through the application of least squares linear regression analysis. Each analyte exhibited good linearity within the range of 50–10,000 ng/mL (R^2^ > 0.99). All samples were measured in triplicate.

### 2.6. Method Validation

The detection limit (LOD) and quantification limit (LOQ) were established using signal-to-noise ratios of 3 and 10, respectively. The recovery of the method was assessed using standard addition. Three different concentration levels of the reference standard (approximately corresponding to matrix concentrations of 100, 500, and 2000 ng/mL) were added to the samples in triplicate. The mean recovery was determined utilizing the subsequent formula: Recovery (%) = (observed value − initial value) × (amount of spike)^−1^ × 100%. Additionally, the relative standard deviation (RSD) was calculated using the formula RSD (%) = (standard deviation/average) × 100%.

### 2.7. Gut Microbiota Analysis

Fecal microbial DNA was extracted using a soil DNA extraction kit (Shenzhen Anbisen Technology Co., Ltd., Shenzhen, China). The V3–V4 hypervariable region of the bacterial 16S rRNA gene was amplified with the primer pair 338F (5′-ACTCCTACGGGAGGCAGCA-3′) and 806R (5′-GGACTACHVGGGTWTCTAAT-3′). The PCR program was set as 95 °C for 5 min, 25 cycles of 95 °C for 30 s, 50 °C for 30 s and 72 °C for 30 s, with a final extension at 72 °C for 7 min. Amplicons were verified by 1.8% agarose gel electrophoresis and purified using the GeneJET Gel Extraction Kit (Thermo Fisher Scientific, Waltham, MA, USA). Qualified libraries were subjected to paired-end sequencing on the Illumina NovaSeq platform by Beijing Baimaike Biotechnology Co., Ltd., Beijing, China. Raw FASTQ reads were quality-filtered using Trimmomatic v0.33, and primer sequences were trimmed with Cutadapt 1.9.1. Paired-end clean reads were assembled via Usearch v10, and chimeric sequences were removed using UCHIME v4.2 to obtain valid sequences. After quality control, assembly, and chimeric element removal, a total of 255,262 valid sequences were obtained from the 10 samples, with an average sequence length of 455.91 bp. Sequences were clustered into OTUs at 97% similarity using Usearch v10.0, and taxonomic annotation of OTUs and ASVs was performed in QIIME2 with a confidence threshold of 70%. All original 16S rRNA sequencing data have been deposited in the NCBI Sequence Read Archive (SRA) under accession number PRJNA1465781.

### 2.8. Statistical Analysis

Pharmacokinetic metrics, such as the area under the plasma concentration–time curve (AUC), terminal elimination half-life (T_1/2_), and mean residence time (MRT), were determined through non-compartmental analysis utilizing DAS 2.0 software. The maximum concentration in plasma (C_max_) and the time at which this concentration peaked (T_max_) were extracted directly from the plasma concentration–time profile. Results are expressed as mean values accompanied by standard deviation (SD). Statistical evaluation of all experimental data employed one-way analysis of variance (ANOVA), with a significance threshold set at *p* < 0.05. Data processing was carried out using GraphPad Prism software, version 10.1.2.

## 3. Results

### 3.1. Validation of the LC-MS/MS Method

Table 1 lists 15 representative calibration data results for ginsenosides Re, Rg1, Rf, Rb1, Rc, Rb2, Rd, Rg2, F1, F2, and compound K in plasma samples used for analysis.

Eleven chemical substances exhibited good linear relationships within their respective concentration ranges, with R^2^ values greater than 0.99, a detection limit of 1.1 to 5.4 ng·mL^−1^, and average recovery rates ranging from 77.8% to 117.0%. The RSD values were less than 12.2% (*n* = 5). The validation confirmed that this LC-MS/MS method can be used for the detection and qualitative and quantitative analysis of 11 ginsenoside compounds in plasma.

### 3.2. Composition Characteristics of the Gut Microbiota in the PGE Group and CK Group

A total of 10 samples (5 per group) were analyzed herein. The V3-V4 region of 16S rRNA genes in rat feces before and after gavage was sequenced via Illumina HiSeq 2500. After chimera filtering, 255,262 sequences were acquired with an average length of 455.91 bp. Each sample generated 23,158–28,674 qualified sequences, with an average of 25,526 reads per sample, which ensured sufficient coverage of the intestinal microbiome. The ACE value, Chao1 value, and Shannon index were used to analyze the microbial α-diversity indices of the CK and PGE groups of rats. The results are shown in Table 2, indicating that the number of OTUs in the PGE group is greater than that in the CK group. The ACE value, Chao1 value, and Shannon index of the CK group were all lower than those of the PGE group, while the Simpson index showed no significant difference. This indicates that long-term oral administration of ginsenosides can lead to an increase in the abundance and diversity of the gut microbiota in rats.

**Figure 1:**
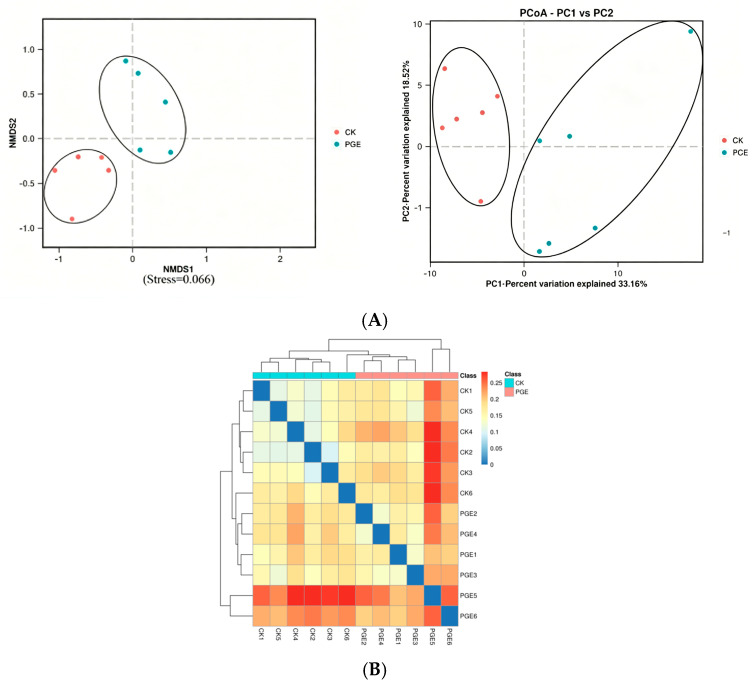

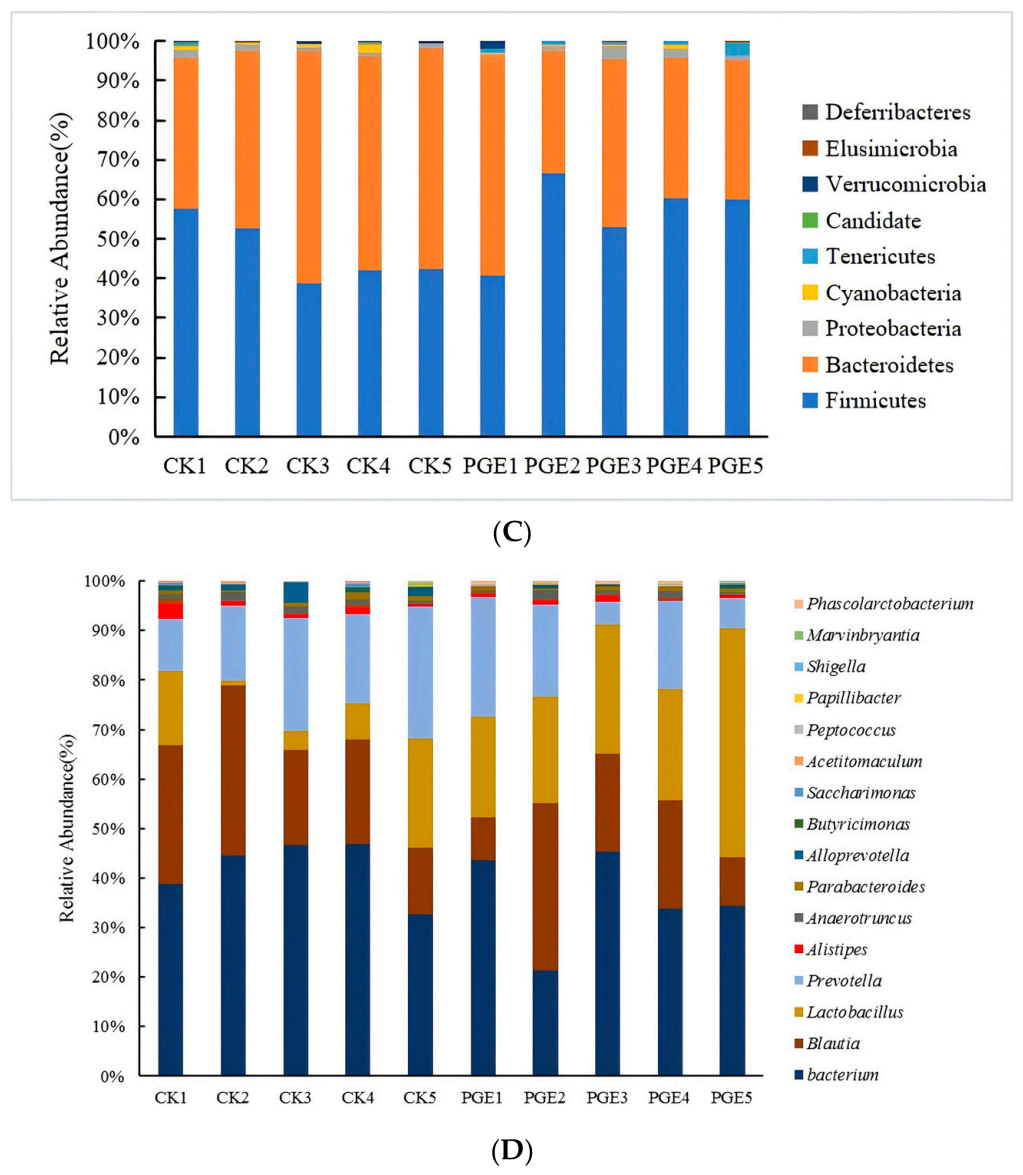
The effect of ginsenosides on the intestinal flora of mice. (**A**) PCoA and NMDS analysis plots. (**B**) Analysis of group microbial community differences based on unweighted uniform score heatmaps. (**C**) Relative abundance of gut microorganisms at the phylum level. (**D**) Relative abundance of gut microorganisms at the genus level.

Analysis of β-diversity revealed clear separation between CK and PGE groups via PCoA and NMDS analyses (stress = 0.066 < 0.2), indicating differences in their microbial community structures (Figure 1A). The unweighted UniFrac distance heatmap, combined with hierarchical clustering, showed that samples from the CK and PGE groups formed two distinct clusters. This clear separation confirms that the two groups have significantly different microbial community structures (Figure 1B).

At the phylum level, *Firmicutes* and *Bacteroidetes* dominated the gut microbiota in both groups. Compared with controls, the PGE treatment increased *Firmicutes* abundance and decreased *Bacteroidetes* abundance, resulting in a higher F/B ratio, while other phyla remained largely unchanged (Figure 1C).

At the genus level, *Bacterium*, *Blautia*, *Lactobacillus*, and *Prevotella* dominated the microbiota. Compared with the control, the PGE treatment significantly increased the relative abundance of *Lactobacillus* while reducing *Blautia* and *Bacterium* (Figure 1D).

### 3.3. Pharmacokinetic Analysis of Ginsenosides in Plasma

**Figure 2:**
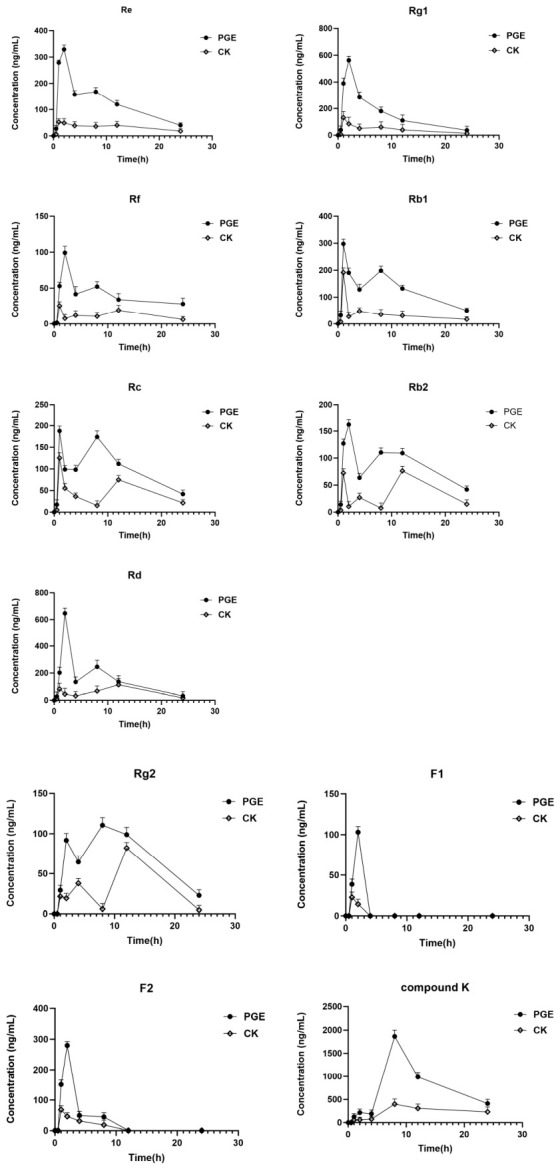
The mean concentration–time curves x±s,n=5 of seven major ginsenosides (Rg1, Re, Rf, Rb1, Rc, Rb2, and Rd) and four rare ginsenosides (ginsenoside Rg2, F1, F2, and CK) in rat plasma.

In order to evaluate the bioavailability of ginsenosides and their metabolites, plasma samples were gathered at various time intervals. This analysis identified a total of 11 target components, comprising 7 primary ginsenosides (Rg1, Re, Rf, Rb1, Rc, Rb2, and Rd) along with 4 metabolites (Rg2, F1, F2, and Compound K). The average concentration–time curves of the 11 components were plotted with plasma concentration on the vertical axis and time on the horizontal axis (Figure 2), with pharmacokinetic parameters shown in Table 3. The area under the plasma concentration–time curve (AUC) and peak concentration (C_max_) of ginsenosides and their metabolites in the PGE group were significantly higher than those in the CK group, indicating a greater relative amount of ginsenosides entering systemic circulation after long-term intervention and better absorption. Mean residence time (MRT), a pivotal parameter reflecting the overall in vivo persistence of drugs, showed no significant change between the two groups, suggesting that the intervention did not alter the average systemic retention profile of ginsenosides. Meanwhile, the delayed Tmax of Rb2, Rd, and Rg2 in the PGE group indicated an altered absorption pattern. The elimination half-life (T_1/2_) is a key indicator for evaluating in vivo drug clearance metabolism. Prolonged T_1/2_ in the CK group indicated suppressed metabolic capacity, which slowed the excretion of ginsenosides and tended to cause in vivo accumulation. In comparison, the markedly shortened T_1/2_ in the PGE group demonstrated that PGE intervention effectively enhanced the metabolic clearance capability, thereby accelerating the elimination of ginsenosides.

### 3.4. Analysis of the Metabolic Pathways of Ginsenosides in Rats

**Figure 3:**
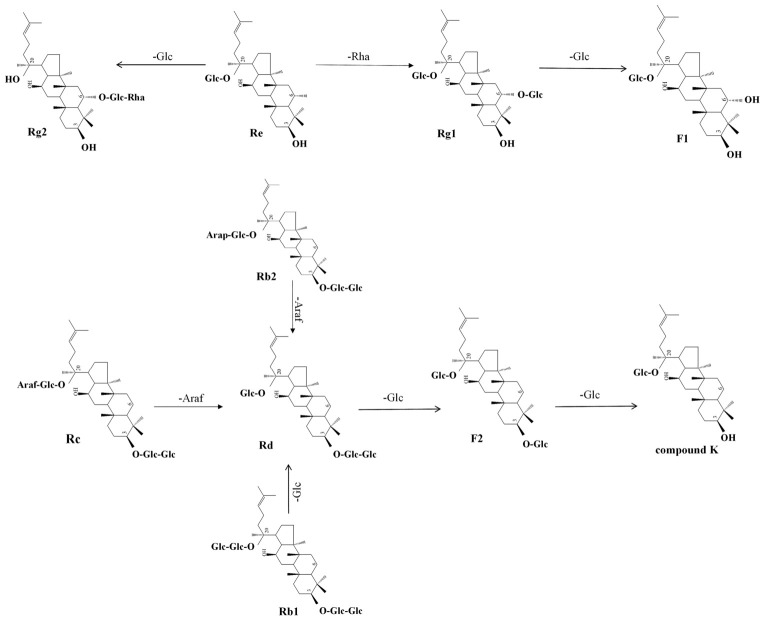
Analysis of the conversion pathway of ginsenoside in rats.

The metabolic pathways of ginsenosides in rats primarily involve a stepwise deglycosylation process mediated by the gut microbiota. According to pharmacokinetic data, the AUC and C_max_ values of Compound K in the PGE group were significantly higher than those in the CK group, indicating greater biotransformation efficiency. In the PGE group, the AUC and C_max_ values of Rd were higher, while those of PPD-type ginsenosides such as Rb1, Rb2, and Rc were relatively lower. This may suggest that under PGE treatment, protopanaxdiol-type ginsenosides are more readily metabolized into Rd and Compound K. Ginsenoside F2 may be formed from the deglycosylation of PPD-type ginsenosides, with the metabolic pathway being Rb1/Rb2/Rc → Rd → F2 → CK. In contrast, F1 and Rg2 may be formed from the deglycosylation of PPT-type ginsenosides, with the metabolic pathways being Re → Rg1 → F1 or Re → Rg2 (Figure 3). In contrast, the AUC and C_max_ values of Compound K in the CK group were significantly lower than those in the PGE group. Conversely, the AUC and Cmax values of protopanaxdiol-type ginsenosides such as Rb1, Rb2, and Rc were relatively high in the CK group, suggesting that the metabolic transformation of protopanaxdiol-type ginsenosides may be slower in the CK group.

## 4. Discussion

The bioavailability and ultimate efficacy of ginsenosides—the main active components of ginseng—after oral administration are not solely determined by the compounds themselves, but are also closely related to the gut microbiota in the human body [25]. Studies have shown that ginsenosides can significantly regulate the composition of the gut microbiota, and this regulatory effect is one of the key mechanisms through which they exert various health benefits, such as immune modulation and liver protection [26,27]. The composition and functional status of the gut microbiota are typically assessed using indicators such as the alpha diversity index and the ratio of *Firmicutes* to *Bacteroidetes* [28]. This study found through animal experiments that continuous intake of ginsenosides for 30 days significantly enhanced the alpha diversity of the gut microbiota. Ginsenosides contain polysaccharide and terpenoid structures, which may provide metabolic substrates for specific microorganisms, promoting the proliferation of beneficial bacteria and the ecological stability of the microbiota [29,30].

At the phylum level, there was an observed increase in the relative abundance of *Firmicutes*, accompanied by a decline in *Bacteroidetes*, resulting in an elevated F/B ratio. This change is consistent with the regulatory patterns of many prebiotics and plant-derived bioactive compounds. Buckwheat polysaccharides (FTP and FEP) increase the F/B ratio and enhance the production of short-chain fatty acids (SCFAs) in the treatment of colitis [31]. Selenium-enriched soluble dietary fiber from corn increased the F/B ratio and the abundance of *Lactobacillus* in DSS-induced colitis mice, thereby alleviating weight loss and colon shortening [32]. This ratio has been recognized as a core biomarker reflecting intestinal microbiota homeostasis and the host’s energy metabolic status. Mechanistically, an increased F/B ratio may correlate with improved host metabolism via lowering serum triglyceride, total cholesterol and blood glucose, and could attenuate intestinal low-grade inflammation. Such microbial shifts might downregulate pro-inflammatory cytokines (TNF-α, IL-6, IL-1β) and elevate anti-inflammatory IL-10, which potentially suppresses intestinal inflammatory infiltration and preserves intestinal mucosal barrier integrity. Notably, several studies have also reported a decrease in the F/B ratio under different intervention doses, treatment durations, and animal models, indicating that the trend of this ratio is easily influenced by experimental conditions. Overall, ginsenosides exhibit significant prebiotic-like effects, capable of optimizing the gut microbial community structure and maintaining intestinal ecological stability.

At the genus level, the proportion of *Lactobacillus* increased, while the proportions of *Blautia* and *Bacterium* decreased. Lactobacillus can secrete β-glucosidase, which is the key enzyme that catalyzes the deglycosylation of ginsenosides into highly active rare saponins, such as compound K [33,34]. Isolated from Korean kimchi, *Levilactobacillus brevis* THK-D437 highly expresses β-glucosidase. Its fermented ginsenosides facilitate hair follicle cell proliferation and ameliorate follicle conditions in mice, showing promising hair growth efficacy [35]. This directly demonstrates that *Lactobacillus* is one of the key microorganisms driving the biotransformation of ginsenosides and enhancing their bioavailability. Furthermore, the enrichment of *Lactobacillus* is functionally linked to host metabolic and inflammatory regulation: this genus can promote the synthesis and secretion of short-chain fatty acids (acetate, propionate, and butyrate), which not only provide energy for intestinal epithelial cells to strengthen intestinal barrier function, but also inhibit the release of systemic inflammatory mediators. *Lactobacillus*-mediated ginsenoside biotransformation also improves lipid metabolism and lowers oxidative stress markers such as malondialdehyde, thereby alleviating host metabolic disorders. There are significant limitations when exploring the functions of lactic acid bacteria solely based on genus-level classification. While *lactobacilli* genera share common metabolic characteristics, their β-glucosidase activity, substrate specificity and metabolic products vary markedly across species and strains. For instance, *Lentilactobacillus buchneri* URN103L isolated from Korean fermented vegetable foods showed potent ginsenoside Rb1 hydrolytic capacity, standing out among 17 screened lactic acid bacteria, whereas congeneric strains lacked such catalytic activity. Homology analysis indicated that despite 99% genetic similarity between URN103L and *Lentilactobacillus buchneri* NRRLB 30929, minor discrepancies in gene clusters and regulatory elements could induce divergent metabolic functions [36]. Accordingly, accurate identification down to species or strain level is essential for ginsenoside biotransformation research to guarantee result reproducibility and application reliability.

The composition and functional status of the gut microbiota are typically assessed using indices such as the α diversity index and F/B ratio, and variations in these indices are closely related to the pharmacokinetic parameters of ginsenosides (such as AUC, Cmax, Tmax, etc.) [37]. The pharmacokinetic results of this study indicate that after long-term intervention, the AUC and Cmax of most ginsenosides and their metabolites in the PGE group were significantly higher than those in the control group, particularly the exposure of ginsenoside Compound K, revealing a higher relative amount of ginsenosides entering the systemic circulation and improved absorption following long-term intervention. This phenomenon may be closely related to remodeling of the structure and function of the gut microbiota: a diverse gut microbiota may provide a more complete and stable metabolic network conducive to the biotransformation of saponins [38]. The enrichment of key functional microbial groups such as *Lactobacillus* may enhance β-glucosidase activity, thereby promoting the hydrolysis and absorption of saponins. Changes in the ratio of *Firmicutes* to *Bacteroidetes* may enhance intestinal barrier function and local absorption efficiency, indirectly facilitating the transmembrane transport of saponins [39,40,41].

Currently, most studies focus on the short-term effects of individual saponin components (such as Rb1 and Rg1) on gut microbiota regulation, while systematic research on the long-term intervention of total saponins is relatively lacking. This study found that long-term oral administration of ginsenosides can alter the composition of the gut microbiota and enhance their bioavailability. Compared to studies that focus solely on individual components or short-term effects, this research better reflects the composite effects observed in practical applications. However, the increase in the ratio of *Firmicutes* in this study is not entirely consistent with the trend of increased *Bacteroidetes* observed in some literature following prebiotic intervention, which may be related to the specificity of ginsenoside structures, differences in animal models, duration of intervention, and detection methods. This reveals that the regulatory effects of functional components with different structures on the gut microbiota may be specific. This study still has limitations. First, the study is based solely on 16S rRNA sequencing for microbiota structural analysis, which fails to reveal the specific changes in functional genes, metabolic pathways, and microbial metabolites, limiting the depth of mechanistic elucidation. Second, the experiment only selected healthy male rats and did not consider the potential effects of sex, age, and metabolic status on the interaction between microbiota and saponins. In future research, we will combine metagenomics and metabolomics with other omics technologies to systematically reveal the effects of ginsenosides on functional modules of the gut microbiota (such as short-chain fatty acid synthesis and bile acid metabolism) and to clarify key metabolic pathways and enzyme systems. We will also increase the sample size and consider animals of different sexes, ages, and disease models to explore the effects of individual differences on the interaction between saponins and the microbiota. At the same time, we will further analyze the roles of intestinal transport proteins and tight junction proteins in the absorption of saponins under the regulation of the gut microbiota, ultimately advancing clinical trials to validate the long-term effects of ginsenosides on gut microecology and pharmacokinetics.

This study preliminarily demonstrated in an animal model that prolonged ginsenoside intake enhances its biotransformation by modulating the gut microbiota composition. Given interspecies differences, these animal results cannot be directly extrapolated to humans. Further multidimensional studies are needed to elucidate the underlying molecular mechanisms and support future exploratory translational research for personalized health regulation.

## 5. Conclusions

This study conducted a 30-day gastric gavage experiment combined with 16S rRNA high-throughput sequencing and pharmacokinetic analysis, revealing that the α-diversity indices (such as ACE, Chao1, and Shannon indices) of the gut microbiota in the PGE group of rats were higher than those in the control group, indicating an enhancement in microbial richness and diversity. At the phylum level, an increase in the relative abundance of *Firmicutes* was noted, while a decrease in *Bacteroidetes* was recorded. Additionally, at the genus level, *Lactobacillus* showed significant enrichment. The pharmacokinetic results indicated that the AUC and Cmax values in the PGE group were significantly elevated, suggesting that alterations in gut microbiota structure facilitate the hydrolysis and absorption of ginsenosides. The study suggests that long-term oral administration of ginsenosides can alter the composition of the gut microbiota and enhance the bioavailability of ginsenosides in the body. However, the research on how changes in gut microbiota composition affect host health is not in-depth; future studies will shift the focus from descriptive phenomena to elucidating the underlying mechanisms.

## Figures and Tables

**Table 1:** Calibration data for the determination of ginsenosides and their metabolites in blood samples.

Ginsenosides	Calibration Curve Equation	Correlation Coefficient	Range (ng·mL^−1^)	LOD (ng·mL^−1^)	LOQ (ng·mL^−1^)	Mean Extraction Recovery (%)		
						100 ng·mL^−1^	500 ng·mL^−1^	2000 ng·mL^−1^
Re	y = 10.691x + 2519.7	R^2^ = 0.9991	50–10,000	2.9	8.8	83.5 (6.4)	72.4 (7.6)	105.6 (12.2)
Rg1	y = 13.074x − 7896.7	R^2^ = 0.9923	50–10,000	2.8	7	88.9 (4.5)	86.5 (7.7)	103.2 (1.1)
Rf	y = 9.781x + 4535.2	R^2^ = 0.9962	50–10,000	5.4	14.1	98.6 (7.8)	84.5 (6.5)	109.9 (9.4)
Rb1	y = 4.788x + 360.2	R^2^ = 0.9948	50–10,000	1.6	4.6	90.5 (4.9)	80.0 (8.5)	109.0 (8.5)
Rc	y = 1.924x + 520.2	R^2^ = 0.9957	50–10,000	2.4	7.4	112.1 (8.4)	104.9 (7.0)	99.1 (6.6)
Rb2	y = 1.635x + 545.7	R^2^ = 0.9929	50–10,000	1.6	4.9	98.1 (9.8)	102.0 (0.7)	106.3 (2.5)
Rd	y = 10.886x + 1745.9	R^2^ = 0.9953	50–10,000	2.1	6.7	96.2 (8.3)	99.5 (0.5)	96.1 (5.0)
Rg2	y = 0.569x + 389.9	R^2^ = 0.9979	50–10,000	3.2	9.1	96.2 (10.0)	95.5 (9.4)	105.5 (9.1)
F1	y = 4.189x + 1495.3	R^2^ = 0.9955	50–10,000	1.4	4.4	78.6 (8.0)	101.6 (9.8)	82.3 (8.6)
F2	y = 11.384x + 4093.2	R^2^ = 0.9951	50–10,000	1.6	4.7	105.0 (8.2)	107.0 (11.5)	117.0 (5.1)
compound K	y = 6.532x + 1551.7	R^2^ = 0.9952	50–10,000	1.1	3.4	88.9 (9.1)	111.0 (1.5)	77.8 (9.6)

**Table 2:** Number of sequences analyzed, Operational Taxonomic Units (OTUs), estimated OTU richness (ACE and Chao1), and diversity (Simpson and Shannon) (*n* = 5).

Groups	Number	Total Reads	Phylotype				
			OTUs	ACE	Chao1	Simpson	Shannon
CK ^a^	CK1	111,684	690	712.4	727.8	0.02	4.7
	CK2	123,179	609	623.5	621.4	0.03	4.5
	CK3	108,419	639	622.6	632.2	0.03	4.5
	CK4	109,482	670	687.6	701.1	0.04	4.7
	CK5	110,604	629	655.5	655.5	0.05	4.1
Mean ± SD		112,674 ± 5365	647 ± 29	660.3 ± 35.4	667.6 ± 40.7	0.03 ± 0.01	4.5 ± 0.2
PGE ^b^	PGE1	102,697	671	700.7	720.4	0.03	4.3
	PGE2	140,182	698	722.9	726.1	0.03	4.5
	PGE3	129,175	700	715.5	720.5	0.03	4.5
	PGE4	118,660	641	662.6	667.8	0.06	4.9
	PGE5	222,227	685	699.9	713.3	0.02	4.6
Mean ± SD		142,588 ± 41,694	679 ± 22	700.3 ± 20.8	709.6 ± 21.3	0.03 ± 0.01	4.6 ± 0.2

^a^ CK, the control group received distilled water intragastrically once daily for 30 days ^b^ PGE, the treatment group received 100 mg/kg of ginsenosides via gastric administration once daily for 30 days.

**Table 3:** The main pharmacokinetic parameters of ginsenoside Re, Rg1, Rf, Rb1, Rc, Rb2, Rd, Rg2, F1, F2 and compound K after oral administration of *Panax ginseng* extract in plasma from the PGE and CK groups (*n* = 5).

Group	Components	T_1/2_ (h)	MRT(h)	AUC (ng·mL^−1^·h^−1^)	Tmax (h)	Cmax (ng·mL^−1^)
PGE	Re	7.8 ± 1.8	8.4 ± 3.7	3070.2 ± 0.6	2.0 ± 0.2	331.4 ± 34.3
	Rg1	5.8 ± 2.4	7.1 ± 2.1	3812.9 ± 0.7	2.0 ± 0.4	562.4 ± 30.5
	Rf	6.6 ± 3.0	10.2 ± 4.0	980.3 ± 0.2	2.0 ± 0.4	99.9 ± 12.4
	Rb1	8.1 ± 1.0	9.4 ± 2.5	3083.1 ± 0.6	1.0 ± 0.6	298.8 ± 49.1
	Rc	7.9 ± 5.0	9.8 ± 2.4	2434.1 ± 0.5	1.0 ± 0.4	188.2 ± 30.9
	Rb2	11.4 ± 3.6	10.2 ± 3.1	2115.8 ± 0.5	2.0 ± 0.4	162.2 ± 24.7
	Rd	4.4 ± 2.6	7.6 ± 2.4	3887.3 ± 0.8	2.0 ± 0.6	648.1 ± 55.1
	Rg2	6.9 ± 4.2	10.1 ± 2.2	1742.7 ± 0.4	8.0 ± 0.4	111.0 ± 24.7
	F1	1.28 ± 1.6	1.8 ± 0.5	184.4 ± 0.1	2.0 ± 1.3	103.5 ± 12.4
	F2	2.5 ± 1.0	3.4 ± 1.0	860.3 ± 0.2	2.0 ± 1.1	280.9 ± 44.7
	compound K	7.5 ± 2.2	11.4 ± 3.6	18,959.9 ± 4.0	8.0 ± 2.0	1889.0 ± 271.2
CK	Re	15.6 ± 2.4	10.2 ± 4.6	810.3 ± 0.7	1.0 ± 0.2	52.3 ± 43.0
	Rg1	7.4 ± 2.2	8.6 ± 2.0	1147.4 ± 0.9	1.0 ± 0.4	144.8 ± 45.0
	Rf	19.7 ± 4.2	10.8 ± 4.3	305.6 ± 0.4	1.0 ± 0.3	25.2 ± 10.4
	Rb1	12.8 ± 1.0	8.8 ± 2.9	820.5 ± 1.1	1.0 ± 0.3	191.5 ± 40.2
	Rc	20.4 ± 3.4	10.7 ± 2.6	1088.4 ± 0.6	1.0 ± 0.5	125.0 ± 35.0
	Rb2	4.1 ± 2.8	11.4 ± 3.2	870.9 ± 0.8	12.0 ± 0.6	76.4 ± 25.6
	Rd	4.2 ± 2.4	10.4 ± 2.0	1843.3 ± 0.8	12.0 ± 0.6	115.2 ± 48.6
	Rg2	5.5 ± 5.0	10.3 ± 3.5	850.76 ± 0.5	12.0 ± 0.3	82.7 ± 20.4
	F1	1.6 ± 0.6	1.5 ± 0.6	38.9 ± 0.1	1.0 ± 1.0	22.5 ± 12.8
	F2	5.1 ± 0.8	7.5 ± 1.1	385.7 ± 0.2	1.0 ± 1.2	68.7 ± 30.8
	compound K	3.7 ± 2.6	13.1 ± 3.4	5630.2 ± 4.0	8.0 ± 1.8	403.0 ± 201.2

## Data Availability

The datasets generated during and/or analyzed during the current study are available from the corresponding author on reasonable request.

## Abbreviations

CK group, control group; PGE group, ginsenoside treatment group; F/B, the ratio of *Firmicutes* to *Bacteroidetes*; AUC, the area under the concentration-time curve; C_max,_ maximum concentration; MRT, mean residence time; T_1/2_, elimination half-life; T_max,_ time to maximum concentration.
